# Safety of flexible bronchoscopy in elderly patients

**DOI:** 10.1186/s12890-026-04326-8

**Published:** 2026-05-06

**Authors:** Stephanie Spiegel, Ralf-Harto Hübner, Dirk Schürmann, Konrad Neumann, Eva Pappe, Thomas Sgarbossa, Martin Witzenrath, Jacopo Saccomanno

**Affiliations:** 1 Department of Infectious Diseases, Respiratory Medicine and Critical Care, Charité – Universitätsmedizin Berlin, Freie Universität Berlin and Humboldt-Universität zu Berlin, Berlin, Germany; 2 Institute of Biometrics and Clinical Epidemiology, Charité – Universitätsmedizin Berlin, Freie Universität Berlin and Humboldt-Universität zu Berlin, Berlin, Germany; 3 CAPNETZ Foundation, Hannover, Germany; 4 German Center for Lung Research (DZL), Giessen, Germany

**Keywords:** Bronchoscopy, Adverse events, Elderly, Patient safety, Risk factors

## Abstract

**Background:**

Flexible bronchoscopy is an indispensable tool in respiratory medicine. In the context of an aging society and increasing life expectancy, the number of elderly, often multimorbid patients is growing. This raises important questions regarding the safety of flexible bronchoscopy in this population.

**Methods:**

In this retrospective study 1841 flexible bronchoscopies performed at two sites of Charité Universitätsmedizin Berlin in the years 2022 and 2023 were assessed and classified into two age groups: patients ≥ 70 years (elderly group) and patients < 70 years (non-elderly group). Safety was assessed by the occurrence of complications, and in a GEE-analysis potential risk factors of complications were identified.

**Results:**

In total, 466 bronchoscopies in the elderly group and 1375 bronchoscopies in the non-elderly group were assessed. Bronchoscopies in the elderly group were performed more frequently under endotracheal intubation than in the non-elderly group (81.8% vs. 70.0%; *p* < 0.001). The overall complication rate was 2.3% with no significant differences between bronchoscopies of the elderly (1.7%) and non-elderly group (2.5%; *p* = 0.345). The most common complications were pneumothorax (0.9%) in the elderly group and hypoxia (0.8%) in the non-elderly group. Transbronchial forceps biopsy (*p* < 0.001; OR = 3.99) and endobronchial valve implantation (*p* = 0.002; OR = 6.44) were significantly associated with an increased risk of complications independent of age.

**Discussion:**

In our study, flexible bronchoscopy proved to be a safe procedure with low complication rates in elderly and non-elderly patients. Age was also not associated with an increased risk of complications. Invasive interventions such as transbronchial forceps biopsies and endobronchial valve implantations were identified as risk factors independent of age. This underscores the importance of individualized risk minimizing strategies, in particular for complex and invasive procedures.

**Supplementary Information:**

The online version contains supplementary material available at 10.1186/s12890-026-04326-8.

## Background

Flexible bronchoscopy is an essential diagnostic and therapeutic procedure in pulmonary medicine. It allows direct visual assessment of the trachea and bronchial system providing access to the lung parenchyma. This enables a wide range of interventions such as bronchoalveolar lavage (BAL), endobronchial ultrasound (EBUS), tissue biopsies, foreign body removal and implantation of stents or valves [1, 2]. Flexible bronchoscopy is a minimal invasive procedure, offers a high diagnostic yield and is therefore indispensable for the diagnosis and treatment of diseases of the respiratory system [3].

In recent years, demographic change and increasing life expectancy have led to a growing population of elderly patients with an increased prevalence of pulmonary diseases [4, 5]. However, advanced age is also associated with physiological changes, which may increase the vulnerability to complications during bronchoscopy. These include reduced functional cardiorespiratory reserves and impaired pharmacodynamic processing of sedatives [6, 7]. In addition, elderly patients often present with multiple comorbidities such as renal insufficiency, frailty or cardiovascular diseases, that can further increase the risk for adverse events [8, 9]. Age- and disease-related factors can alter responses to sedation, airway manipulation and transient hypoxemia and magnify the clinical consequences of complications [9, 10]. At the same time, bronchoscopy is often of particular importance in older patients, as common indications such as suspected malignancy or mediastinal and hilar lymphadenopathy are highly prevalent in this age group [11–13]. Thus, balancing the clinical necessity of bronchoscopy against its potential risks is of central relevance in elderly patients.

The current evidence is based on heterogeneous studies, of which several are limited by small sample sizes (20–39 cases in the elderly) or restricted to specific procedures such as endobronchial ultrasound-guided transbronchial needle aspiration (EBUS-TBNA) [14–16]. Other investigations have reported an increased incidence of certain complications in older patients, including bleeding and pneumothorax [12, 17, 18]. Consequently, the question persists whether age itself meaningfully heightens procedural risk.

For this reason, the aim of this study is to investigate the safety of flexible bronchoscopy in elderly patients in comparison to a younger control group and to evaluate independent risk factors for complications in a large cohort.

## Materials and methods

**Fig. 1:**
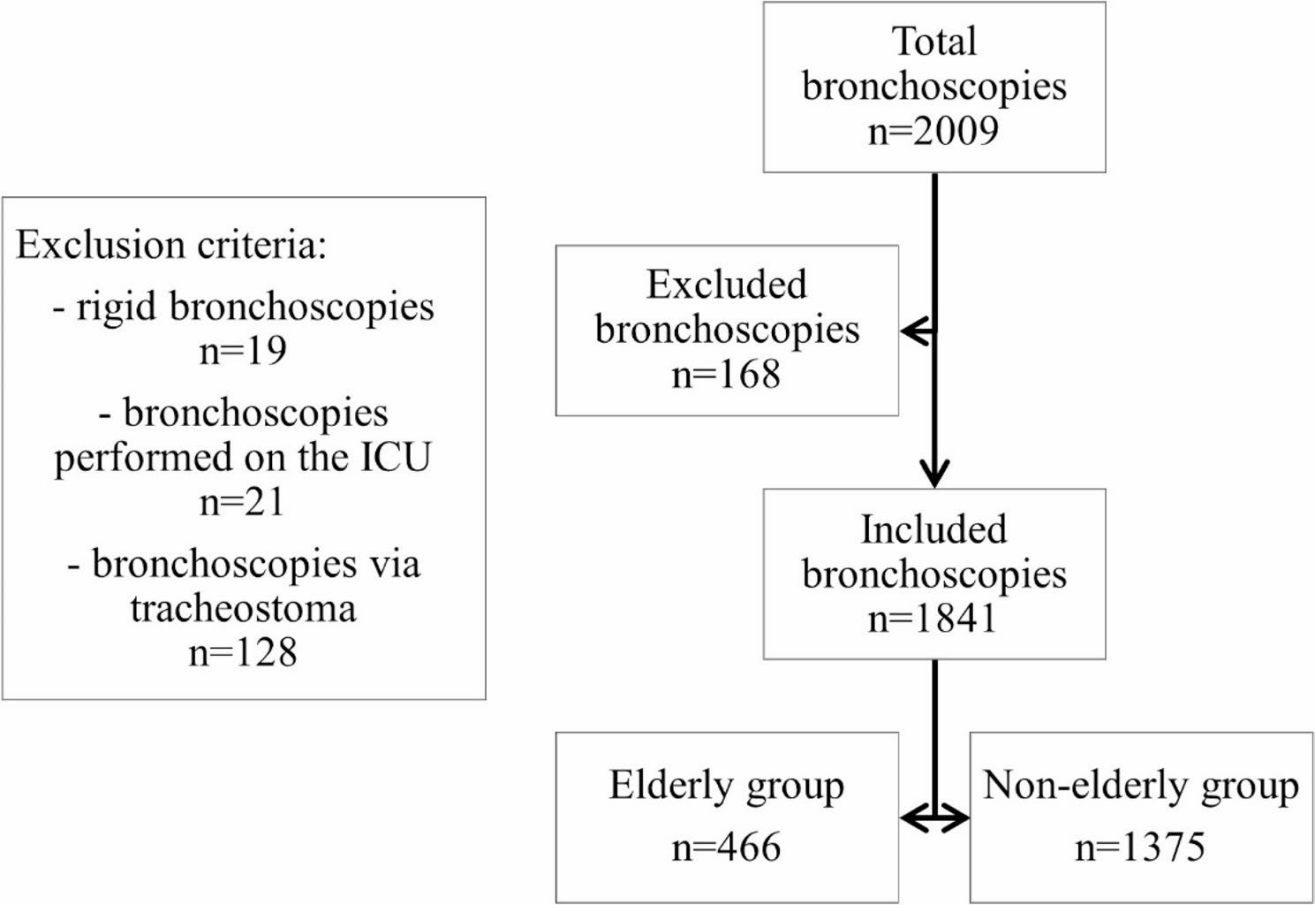
Flowchart selection of bronchoscopies. Out of 2009 bronchoscopies, 168 were excluded based on predefined criteria. A total of 1841 bronchoscopies were included and subsequently stratified by age into two groups: 1375 patients aged < 70 years (non-elderly) and 466 patients aged ≥ 70 years (elderly). N, number of bronchoscopies; ICU, intensive care unit

In this retrospective study, we analyzed 2009 bronchoscopies from two sites of Charité Universitätsmedizin Berlin—Campus Charité Mitte and Campus Virchow Klinikum—covering the years 2022 and 2023. The trial was approved by the local ethics committees of the study centers (EA1/136/24). The examination of bronchoscopies included baseline characteristics, in- or outpatient status, types of airway management, sedation rates, interventions and safety. All flexible bronchoscopies performed during the study period were included, irrespective of whether they were conducted without securing the airway or with an endotracheal tube as airway protection. Rigid bronchoscopies, bronchoscopies performed via tracheostoma and bronchoscopies carried out on the intensive care unit were not included in this analysis. Based on these criteria, we excluded 168 bronchoscopies and formed two groups: bronchoscopies performed on patients that were 70 years or older (elderly group) and bronchoscopies performed on patients younger than 70 years (non-elderly group) at the time of the procedure (see Fig. 1). We defined elderly patients as those aged ≥ 70 years, as this threshold is widely used in clinical research and is epidemiologically relevant given that the median age at lung cancer diagnosis in Germany is approximately 70 years [11, 19, 20]. Furthermore, age-related changes in respiratory structure and function become more pronounced at this age [7, 21]. Several studies evaluating bronchoscopy in older patients have also used a similar threshold, thereby facilitating comparability with existing literature [14, 15, 22].

### Classification of complications

Safety was assessed by the occurrence of complications documented in procedure reports and patient records. Complications were classified into major and minor complications, or death. Major complications were defined as events necessitating a transfer to a higher level of care. The duration of this stay was also recorded. Complications were further classified into the following categories: bleeding, hypoxia, cardiac arrhythmia, hypotonia, pneumothorax, extubation-related complications and others. Hypertension, defined by the application of antihypertensive medication, was also assessed, but did not occur in this cohort and was therefore not reported. Hypotension was defined as requiring administration of sympathomimetic medication. Bleeding events were assessed according to the Nashville Bleeding Scale. The scale classifies bleeding severity from mild, self-limiting bleeding (grade 1) to moderate bleeding requiring endoscopic therapy (grade 2), severe bleeding with hemodynamic instability (grade 3), and life-threatening or fatal hemorrhage (grade 4). Grades 1 and 2 were not reported if they were controlled by standard measures without further clinical impact [23]. Hypoxia was defined as initiation or escalation of ventilatory support due to decrease in oxygen saturation and was graded according to the highest level of support required: Grade I, escalation of supplemental oxygen without ventilatory assistance; Grade II, requirement for non-invasive ventilatory support; Grade III, manifest respiratory failure requiring invasive ventilatory support. Cardiac arrhythmia included cardiac arrest and arrhythmia, that required treatment during the procedure. Extubation-related complications encompassed the need for non-invasive ventilation, delayed awakening and respiratory compromise following extubation. In cases where multiple complications occurred, only the most clinically significant event per patient was recorded, in order to provide a clear and unbiased assessment of complication rates.

### Bronchoscopy procedures

Informed consent for the bronchoscopy was obtained at least 24 h in advance, with the exception of emergency procedures.

In the bronchoscopy suite, patients were positioned in a supine position and it was ensured that an intravenous catheter was available. Bronchoscopies were performed by at least one specialist in respiratory medicine with training in interventional pneumology, assisted by one procedural nurse and one nurse certified in sedation administration. A pre-procedural team time-out was conducted to confirm patient identity, indication for the procedure, allergies and potential risk factors.

The bronchoscopist determined the procedural technique and airway management strategy based on a pre-procedural assessment of comorbidities, baseline oxygenation, airway anatomy, aspiration risk and complexity and duration of the planned interventions [24]. At our institution, airway protection is instituted at a low threshold, with a preference for endotracheal intubation to ensure patient safety.

During the procedure, oxygen saturation, respiratory rate and electrocardiogram (ECG) were continuously monitored and blood pressure was measured at least every three minutes. The patient received oxygenation over a nasal cannula to prevent hypoxia during the bronchoscopy. In most cases, a combination of midazolam and propofol was administered intravenously for procedural sedation. Topical anesthesia was applied stepwise to the patient’s upper airway, the laryngeal inlet, the trachea and endobronchially. When adequate sedation was achieved and no further airway management was necessary, the flexible bronchoscope was introduced transnasally or transorally under direct visualization of the vocal cords and then advanced into the trachea. In cases where the airway was secured a 7.5–8.5 mm endotracheal tube (Bronchoflex; Rüsch GmbH, Rems-Murr, Germany) was inserted into the trachea [25–27]. When required, mechanical ventilation was administered via endotracheal tube with settings adjusted to maintain adequate oxygenation and ventilation during the procedure.

Following completion of the procedure, patients remained under observation until stable vital parameters and full orientation were ensured.

### Statistical analysis

Based on the expected cohort size of approximately 2000 patients and an anticipated complication rate below 5%, the study provides > 80% power (one-sided exact binomial test, α = 0.025) to demonstrate that the true complication rate is below 6.5%. Frequency analysis was performed on nominal variables. Descriptive statistics were calculated to evaluate continuous variables, including mean with standard deviation. Sedation rates and age were compared between the groups using independent samples t-test. Analyses were performed using available data, and cases with missing values were excluded from the respective analyses. The comparison of baseline characteristics, such as gender, status and airway management, interventions and complications between the two groups was analyzed via Pearson’s Chi-square test or Fisher’s Exact Test (< 5 samples). To investigate risk factors for bronchoscopy-associated complications, we performed a generalized estimating equations (GEE)-analysis with a binary logistic link, accounting for repeated procedures within patients. To maintain sufficient statistical power, a cutoff of > 10 cases per intervention and age group was applied for the GEE-analysis. Statistical analyses were conducted using SPSS software (version 29.0.0.0; IBM, Armonk, NY, USA). The statistical significance level was set at 0.05.

## Results

### Baseline characteristics

In the years 2022 and 2023, 1392 patients underwent 1841 flexible bronchoscopies. 196 patients underwent multiple bronchoscopies (up to 19). 466 bronchoscopies (25.3%) were performed in the elderly group and 1375 bronchoscopies (74.7%) the non-elderly group. In the elderly group, the mean age was 75.9 ± 4.7 years and 54.0 ± 11.8 years in the non-elderly group (*p* < 0.001). There was no significant difference in gender distribution (see Table 1).

**Table 1:** Baseline characteristics

	Elderly (*n* = 466)	Non-elderly (*n* = 1375)	*p*-value
Age			**< 0.001**
Mean + SD	75.9 ± 4.7	54.0 ± 11.8	
Minimum-Maximum	70–90	15–69	
Gender, *n* (%)			0.434
Female	211 (45.3%)	594 (43.2%)	
Male	255 (54.7%)	781 (56.8%)	
Patient status, *n* (%)			0.724
Inpatient	392 (84.1%)	1147 (83.4%)	
Outpatient	74 (15.9%)	228 (16.6%)	
Airway management, *n* (%)			**< 0.001**
Intubated	381 (81.8%)	962 (70.0%)	
Non-intubated	85 (18.2%)	413 (30.0%)	

Bold *p*-values indicate statistically significant differences and are highlighted for better readability

*SD* Standard deviation, *N* Number of bronchoscopies

84.1% of bronchoscopies in the elderly group and 83.4% in the non-elderly group were performed on inpatients. In the elderly group, a significantly higher rate of bronchoscopies (81.8%) was performed under endotracheal intubation than in the non-elderly group (70.0%; *p* < 0.001).

### Interventions

**Fig. 2:**
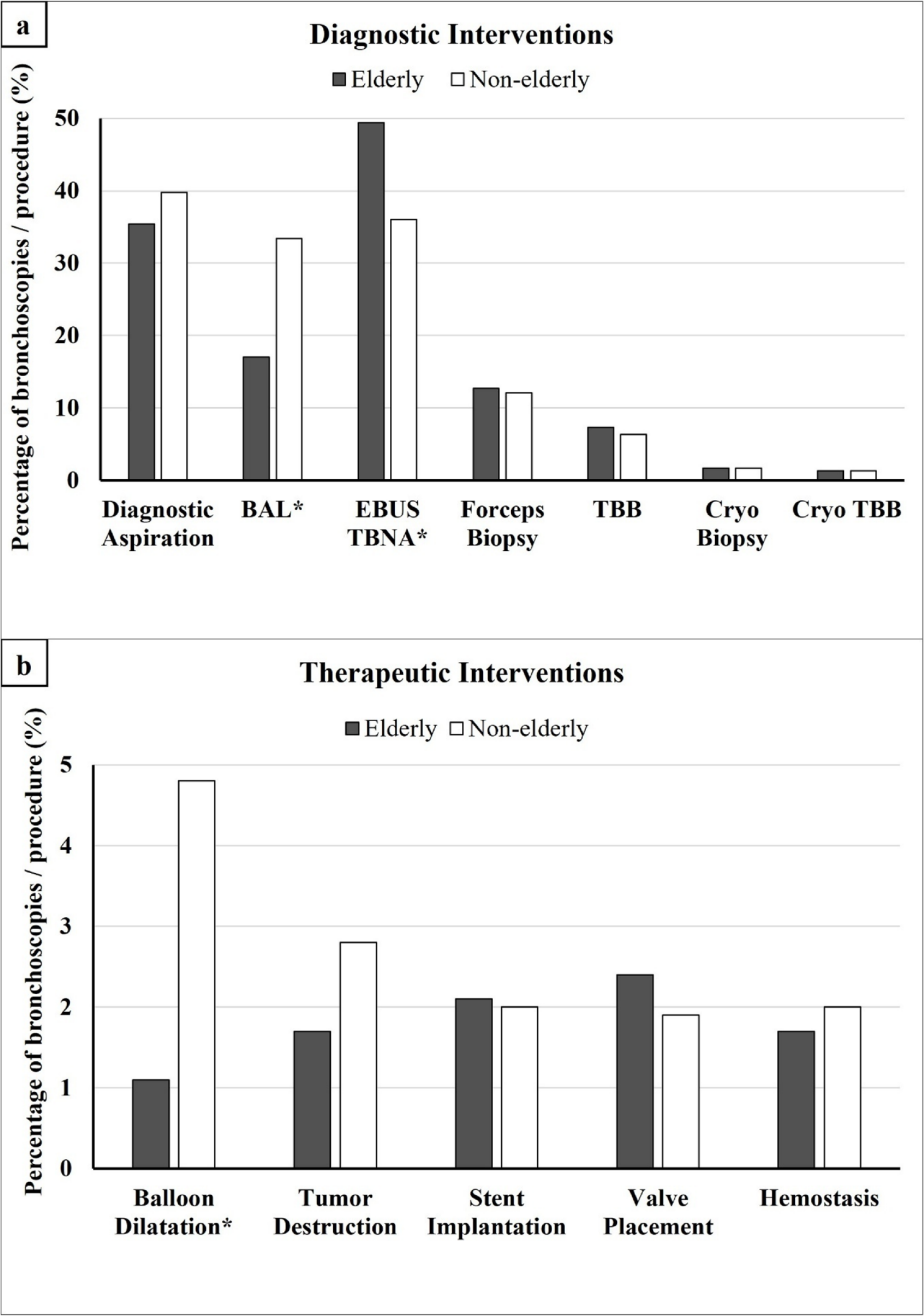
**a** Distribution of diagnostic interventions. Percentages of diagnostic interventions, each calculated relative to the total number of individuals within the respective age group (elderly vs. non-elderly). Asterisks (*) indicate statistically significant differences between the groups. BAL, bronchoalveolar lavage; EBUS TBNA, endobronchial ultrasound-guided transbronchial needle aspiration; TBB, transbronchial forceps biopsy. **b** Distribution of therapeutic interventions. Percentages of therapeutic interventions, each calculated relative to the total number of individuals within the respective age group (elderly vs. non-elderly). Asterisks (*) indicate statistically significant differences between the groups

Interventions are presented as diagnostic and therapeutic interventions in Table 2 and their distribution is shown in Fig. 2a and 2b.

**Table 2:** Diagnostic and therapeutic interventions

	Elderly (*n* = 466)	Non-elderly (*n* = 1375)	*p*-value
Diagnostic interventions, *n* (%)			
Diagnostic aspiration	165 (35.4%)	547 (39.8%)	0.094
BAL	79 (17.0%)	459 (33.4%)	**< 0.001**
EBUS-TBNA	230 (49.4%)	495 (36.0%)	**< 0.001**
Central forceps biopsy	59 (12.7%)	166 (12.1%)	0.738
Transbronchial forceps biopsy	34 (7.3%)	86 (6.3%)	0.431
Central cryobiopsy	8 (1.7%)	23 (1.7%)	0.701
Transbronchial cryobiopsy	6 (1.3%)	18 (1.3%)	0.972
Therapeutic interventions *n* (%)			
Balloon dilatation	5 (1.1%)	66 (4.8%)	**< 0.001**
Tumor destruction	8 (1.7%)	38 (2.8%)	0.211
Stent implantation	10 (2.1%)	28 (2.0%)	0.886
Endobronchial valve implantation	11 (2.4%)	26 (1.9%)	0.532
Hemostasis	8 (1.7%)	28 (2.0%)	0.667
Other Interventions*, *n* (%)	6 (1.3%)	36 (2.6%)	0.138

Bold *p*-values indicate statistically significant differences and are highlighted for better readability

*N* Number of bronchoscopies

^*^Other interventions: foreign body removal, valve removal, ablation cryoprobe, coil placement, cryo EBUS TBNA, Chartis, AeriSeal, stent removal

Among diagnostic procedures, BAL was conducted in 17.0% of cases in elderly patients and in 33.4% of cases non-elderly patients (*p* < 0.001). Endobronchial ultrasound-guided transbronchial needle aspiration (EBUS-TBNA) was carried out in 49.4% of procedures in the elderly group and in 36.0% in the non-elderly group (*p* < 0.001).

There were no statistically significant differences between the groups observed for diagnostic aspiration (35.4% elderly vs. 39.8% non-elderly), forceps biopsy on central lesions (12.7% elderly vs. 12.1% non-elderly), transbronchial forceps biopsy (TBB) (7.3% elderly vs. 6.3% non-elderly), central cryobiopsy (1.7% elderly vs. 1.7% non-elderly) and transbronchial cryobiopsy (1.3% in both groups).

Regarding therapeutic interventions, balloon dilatation was performed in 1.1% of bronchoscopies in the elderly group and in 4.8% of bronchoscopies in the non-elderly group (*p* < 0.001). Other therapeutic interventions showed no statistically significant differences between the age groups including tumor destruction (1.7% elderly vs. 2.8% non-elderly), stent implantation (2.1% elderly vs. 2.0% non-elderly), endobronchial valve treatment (2.4% elderly vs. 1.9% non-elderly) and hemostatic interventions (1.7% elderly vs. 2.0% non-elderly).

The remaining interventions with less than 5 procedures per group were summarized as other diagnostic and therapeutic interventions (1.3% elderly vs. 2.6% non-elderly; *p* = 0.138). These included foreign body removal, endobronchial valve removal, ablation with cryoprobe, coil placement, transbronchial mediastinal cryobiopsy, Chartis measurement, AeriSeal and stent removal.

### Sedation

For sedation midazolam, propofol and ketamine were administered and mostly used in combination. The most frequent regimen was a combination of midazolam and propofol in 96.9% of bronchoscopies. Midazolam was administered in > 96% of procedures in elderly patients and non-elderly patients. However, the mean dose of midazolam was significantly higher in the non-elderly group (4.8 ± 2.1 mg) compared to the elderly group (4.1 ± 1.7 mg; *p* < 0.001). Similarly, propofol was administered in > 97% cases in both groups (see Table 3).

**Table 3:** Sedation

	Elderly (*n* = 466)	Non-elderly (*n* = 1375)	*p*-value
Midazolam			**< 0.001**
Cases, *n* (%)	451 (97.8%)	1342 (97.6%)	
Mean + SD (mg)	4.1 ± 1.7	4.8 ± 2.1	
Propofol			**< 0.001**
Cases, *n* (%)	456 (97.9%)	1327 (96.5%)	
Mean + SD (mg)	163.8 ± 103.4	202.2 ± 133.6	
Ketamine			0.169
Cases, *n* (%)	39 (8.4%)	150 (10.9%)	
Mean + SD (mg)	18.7 ± 9.5	21.8 ± 13.3	

Bold *p*-values indicate statistically significant differences and are highlighted for better readability

*SD* Standard deviation, *N* Number of bronchoscopies

The mean dose was significantly higher in the non-elderly group (202.2 ± 133.6 mg) as compared to the non-elderly group (163.8 ± 103.4 mg; *p* < 0.001). Ketamine was administered in 8.4% of bronchoscopies in the elderly group and in 10.9% of procedures in non-elderly patients (*p* = 0.169).

### Complications

In total, 42 complications during 1841 bronchoscopies were recorded, corresponding to an overall rate of 2.3%. Complications occurred in 1.7% of bronchoscopies in the elderly group and in 2.5% in the non-elderly group with no statistically significant difference between the age groups. As shown in Table 4, the distribution of major and minor complications was likewise comparable across both age groups.

**Table 4:** Major and minor complications

	Elderly (*n* = 466)	Non-elderly (*n* = 1375)	*p*-value
Total complications, *n* (%)	8 (1.7%)	34 (2.5%)	0.345
Death during bronchoscopy	0 (0%)	1 (0.1%)	n.a.
Major complications^a^	5 (1.1%)	15 (1.1%)	0.974
Duration stay on transferred ward (mean days ± SD)	12.2 ± 10.5	4.2 ± 1.4	0.489
Minor complications	3 (0.6%)	18 (1.3%)	0.318
Categorized total complications, n (%)			
Cardiac arrhythmia	0 (0%)	2 (0.1%)	n.a.
Hypoxia	0 (0%)	11 (0.8%)	n.a.
Grade 1	0 (0%)	6 (0.4%)	
Grade 2	0 (0%)	2 (0.1%)	
Grade 3	0 (0%)	3 (0.2%)	
Hypotonia	1 (0.2%)	0 (0%)	n.a.
Bleeding^b^	2 (0.4%)	8 (0.6%)	1.0
Grade 2	1 (0.2%)	2 (0.1%)	
Grade3	1 (0.2%)	6 (0.4%)	
Pneumothorax	4 (0.9%)	4 (0.3%)	0.118
Extubation-related complications	1 (0.2%)	6 (0.4%)	0.687
Other complications^c^	0 (0%)	2 (0.1%)	n.a.

^a﻿^Major complications are defined as events requiring higher level of care

^b﻿^Grade 1 bleeding events were not recorded, as they were self-limiting without clinical impact. Grade 2 events were included if not controlled by standard measures or associated with clinical impact; grade 4 did not occur

^c﻿^Other complications: laryngeal edema, pneumopericardium and pneumomediastinum. *N* Number of bronchoscopies; *n.a*., Not applicable

In the elderly cohort, the most frequently reported complication was pneumothorax (0.9%), followed by bleeding (0.4%), extubation-related complications (0.2%) and hypotonia (0.2%). Bleeding events in the elderly cohort were further classified according to severity as grade 2 (*n* = 1, 0.2%) and grade 3 (*n* = 1, 0.2%).

In the non-elderly cohort, hypoxia (0.8%) was the most common complication, followed by bleeding (0.6%), extubation-related complications (0.4%), pneumothorax (0.3%), cardiac arrhythmia (0.1%) and other complications (0.1%). Hypoxia was further stratified into grade I (*n* = 6, 0.4%), grade II (*n* = 2, 0.1%), and grade III (*n* = 3, 0.2%). Bleeding events were classified as grade 2 (*n* = 2, 0.1%) and grade 3 (*n* = 6, 0.4%).

The distribution of reported complications showed no significant differences between the age groups. There was no complication reported as a direct result of intubation.

One fatality (0.1%) occurred during high-risk bronchoscopy to diagnose an advanced non-small cell lung cancer in the non-elderly cohort due to hypoxia-related pulseless electrical activity after a recent history of fulminant pulmonary embolism.

### Risk factors

To identify potential risk factors for complications associated to bronchoscopy, a Generalized Estimating Equations (GEE)-analysis was performed. Patient characteristics including age group, gender, and patient status were included, along with diagnostic and therapeutic interventions. The threshold of included interventions was set at > 10 times per age group to ensure statistical power (excluded interventions: central cryobiopsy, transbronchial cryobiopsy, balloon dilatation, tumor destruction, stent implantation, hemostasis and other interventions). For this analysis, the elderly cohort was further stratified into two subgroups (70–79 years and ≥ 80 years) to explore potential age-related effects within the elderly population.

Age groups, gender, and patient status (inpatient vs. outpatient) were not significantly associated with an increased risk of complications in bronchoscopies. In contrast, transbronchial forceps biopsy was identified as risk factor of complications (*p* < 0.001, OR = 3.99, 95% CI = 1.77–9.02) as was endobronchial valve implantation (*p* = 0.002, OR = 6.44, 95% CI = 1.92–21.19). Other interventions, including EBUS-TBNA, diagnostic aspiration, BAL, and central forceps biopsy, were not linked to an elevated risk of adverse events (see Table 5).

**Table 5:** Generalized estimating equations-analysis – risk factors for bronchoscopy-associated complications

	*p*-value	Odds Ratio	95%-CI (OR)
Gender	0.760	1.11	0.58–2.10
Patient status	0.318	1.73	0.59–5.05
Age group 70–79	0.125	0.44	0.16–1.25
Age group ≥ 80	0.408	1.56	0.54–4.49
Diagnostic aspiration	0.810	0.92	0.49–1.75
BAL	0.947	0.97	0.47–2.03
EBUS-TBNA	0.983	0.99	0.49–2.01
Central forceps biopsy	0.752	1.16	0.45–2.98
Transbronchial forceps biopsy	**< 0.001**	3.99	1.77–9.02
Endobronchial valve implantation	**0.002**	6.44	1.95–21.19

Procedures with > 10 cases per age group were included in this analysis. Bold *p*-values indicate statistically significant differences and are highlighted for better readability

*OR* Odds ratio, *CI* Confidence interval, *BAL* Bronchoalveolar lavage, *EBUS-TBNA* Endobronchial ultrasound-guided transbronchial needle aspiration

## Discussion

This large retrospective analysis of 1841 flexible bronchoscopies performed on 1392 patients demonstrates that bronchoscopy is a safe procedure with an overall complication rate of 2.3%, when being performed in a large tertiary care center with dedicated bronchoscopy units. Importantly, this low rate was observed despite the wide range of diagnostic and therapeutic interventions. Complication rates did not differ significantly between bronchoscopies in elderly (1.7%) and non-elderly patients (2.5%). In addition, the distribution of major and minor complications was also comparable between the age groups, indicating that advanced age alone is not a risk factor. Instead, higher complication risks were associated with specific procedures such as transbronchial forceps biopsy and endobronchial valve implantation.

Prior studies consistently report that flexible bronchoscopy is a safe procedure in elderly patients and align with our findings [12, 14, 15, 17, 18, 22]. Even though certain investigators found higher frequencies of complications in elderly patients such as bleeding in up to 3.8-4.0% and pneumothorax in up to 3.4%, the overall safety profile remained favorable [12, 17, 18]. However, these studies differ in terms of study design, definition of elderly patients and classification of complications limiting comparability. Moreover, reported complication rates vary considerably across existing literature. Okachi et al., Evison et al. and Yildizeli et al. analyzed the safety of EBUS-TBNA and reported complication rates of 2.9%, 5.1% and 7.7%, respectively, in patients aged 70 years and older [14, 15, 22]. In contrast to our study with a complication rate of only 1.7% in bronchoscopies of elderly patients, these studies focused exclusively on EBUS-TBNA. Additionally, bronchoscopies were performed in light sedation without endotracheal intubation, which may explain the observed differences [14, 15, 22]. The study of Evison et al. with a complication rate of 5.1% focused on patients diagnosed with primary lung cancer. This highly vulnerable population is typically characterized by a higher comorbidity burden related to the underlying condition, that may also affect complication rates [22].

The lower complication rate in elderly patients in our study compared to the above mentioned publications may be explained by several factors. In this cohort, a wide range of diagnostic and therapeutic interventions, including procedures of varying complexity relative to EBUS, were examined. Furthermore, the administered mean doses of propofol and midazolam were significantly lower in the elderly, due to the age-related differences in metabolization of sedatives in elderly patients in contrast to younger patients [10, 28]. Moreover, elderly patients were intubated more frequently than younger patients. Endotracheal intubation provides stable subglottic airway access, facilitates effective suctioning and ensures safe performance of complex procedures [24]. Therefore, it potentially reduced the risk of hypoxemia and aspiration during frequent interventions like EBUS-TBNA. Another noteworthy benefit of endotracheal intubation is higher diagnostic yield for EBUS-TBNA under deep sedation and airway protection reported in a study by Yarmus et al. [29]. Notably, our study reports that EBUS-TBNA was performed more frequently in the elderly group with a favorable safety profile.

The reported differences of BAL and EBUS-TBNA between the age groups likely reflect variations in the underlying clinical indications for bronchoscopy. While suspected malignancy is more common in elderly patients, bronchoscopy in younger patients may be performed more frequently to evaluate interstitial lung diseases and infectious conditions, which often require BAL [12, 30].

Our findings implicate that age itself does not increase the risk of complications during bronchoscopy. However, our generalized estimated equations analysis showed that transbronchial forceps biopsy and endobronchial valve implantation were associated with a higher risk of adverse events. This observation aligns with the invasive nature of both interventions. They should only be performed under certain indications because of their higher risk for bleeding and/or pneumothorax [31–34]. This indicates that individualized procedural strategies regardless of age are necessary to provide safe procedures. It should be noted that transbronchial cryobiopsies, carrying a comparatively high risk for complications such as bleeding and pneumothorax, were not included in the GEE-analysis due to limited number of cases [35].

The higher use of endotracheal intubation in elderly patients raises clinical considerations. While routine prophylactic intubation may not be necessary, maintaining a lower threshold for airway protection may be beneficial in selected patients undergoing complex procedures. In routine practice, endotracheal intubation is more frequently used in bronchoscopic procedures with higher procedural complexity or an increased risk of bleeding, where airway protection facilitates the management of potential complications. Current guidelines already recommend to secure the airway in flexible bronchoscopies that carry a high risk of severe bleeding or involve complex interventions to facilitate adequate management of potential complications and ensure optimal patient safety [24]. However, further studies are needed to better define which patients benefit most from endotracheal intubation.

This retrospective descriptive analysis represents one of the largest cohorts reported to date examining the safety of flexible bronchoscopy in elderly patients. The study population represents a diverse and broad variety of pulmonary pathologies and bronchoscopic procedures performed at a high-volume-tertiary care center. This real-world setting reflects routine clinical practice and therefore strengthens the external validity of our findings. Furthermore, we applied a GEE-based statistical model that accounted for patient characteristics and interventions ensuring a robust and reliable risk factor analysis.

However, certain limitations need to be considered. Due to the retrospective and monocentric design of this study, the results may not be generalizable and carry potential risks for documentation bias. As complications are more likely to be expected in the elderly cohort, there is no indication that reporting bias occurred more frequently in this patient group.

In addition, this study is subject to selection bias. Only patients who ultimately underwent bronchoscopy were included, so individuals who were not referred due to frailty, clinical instability, or patient preference are not captured. Furthermore, structured assessment of patient-related risk factors such as ASA classification, frailty scores, pre-existing comorbidities, and procedure duration was not available [9, 36, 37]. Together, these limitations may lead to underrepresentation of frailer elderly patients and underestimation of procedural risk in the elderly population. Nevertheless, our cohort is heterogeneous and includes a substantial proportion of patients with higher risk and relevant comorbidities, while all patients received individual risk assessment, guideline-based precautions, and airway management.

Furthermore, the lack of structured follow-up data limits the analysis to peri-procedural outcomes and precludes assessment of potential long-term consequences.

Considering the cohort imbalance and the low number of complication events, the possibility of clinically relevant differences cannot be fully ruled out. Therefore, further prospective multicenter studies are necessary to confirm our findings and refine risk stratification strategies for elderly patients undergoing bronchoscopy. In particular, studies that focus on optimizing airway management strategies and long-term outcomes following bronchoscopy would be of high clinical relevance.

## Conclusion

In our study, flexible bronchoscopy under sedation was safe across all groups with low complication rates. While our findings indicate that invasive interventions, such as transbronchial forceps biopsies and endobronchial valve implantations, are linked to a higher risk of adverse events, age did not heighten procedural risk. Careful patient selection, guideline-based safety protocols, and individualized procedural strategies, including tailored sedation and airway management, remain key elements for ensuring procedural safety in an increasingly elderly patient population. 

## Supplementary Information

Supplementary Material 1.

## Data Availability

The datasets used and analyzed during the current study are available from the corresponding author on reasonable request.
