# Supplementary material for: Safety of flexible bronchoscopy in elderly patients
Source: BMC Pulm Med. 2026 May 6;26:211. doi: 10.1186/s12890-026-04326-8 (PMC13151341; doi:10.1186/s12890-026-04326-8)
Supplement: Supplementary file 1 — Supplementary Material 1. [file 12890_2026_4326_MOESM1_ESM.docx]

STROBE Statement—checklist of items that should be included in reports of observational studies

|  | Item No. | Recommendation | Page  No. | Relevant text from manuscript |
| --- | --- | --- | --- | --- |
| **Title and abstract** | 1 | (*a*) Indicate the study’s design with a commonly used term in the title or the abstract | 2 | In this retrospective study 1841 flexible bronchoscopies performed at two sites of Charité Universitätsmedizin Berlin in the years 2022 and 2023 were assessed and classified into two age groups: patients ≥70 years (elderly group) and patients <70 years (non-elderly group). |
|  |  | (*b*) Provide in the abstract an informative and balanced summary of what was done and what was found | 2 | Safety was assessed by the occurrence of complications, and in a GEE-analysis potential risk factors of complications were identified. […] The overall complication rate was 2.3% with no significant differences between bronchoscopies of the elderly (1.7%) and non-elderly group (2.5%; p=0.345). […] Transbronchial forceps biopsy (p<0.001; OR=3.99) and endobronchial valve implantation (p=0.002; OR=6.44) were significantly associated with an increased risk of complications independent of age. |
| Introduction | | | |  |
| Background/rationale | 2 | Explain the scientific background and rationale for the investigation being reported | 4-5 | The current evidence is based on heterogeneous studies, of which several are limited by small sample sizes (20-39 cases in the elderly) or restricted to specific procedures such as endobronchial ultrasound-guided transbronchial needle aspiration (EBUS-TBNA) (14–16). […] Consequently, the question persists whether age itself meaningfully heightens procedural risk. |
| Objectives | 3 | State specific objectives, including any prespecified hypotheses | 4 | The aim of this study is to investigate the safety of flexible bronchoscopy in elderly patients in comparison to a younger control group and to evaluate independent risk factors for complications in a large cohort. |
| Methods | | | |  |
| Study design | 4 | Present key elements of study design early in the paper | 5 | In this retrospective study, we analyzed 2009 bronchoscopies from two sites of Charité Universitätsmedizin Berlin—Campus Charité Mitte and Campus Virchow Klinikum—covering the years 2022 and 2023. |
| Setting | 5 | Describe the setting, locations, and relevant dates, including periods of recruitment, exposure, follow-up, and data collection | 5 | In this retrospective study, we analyzed 2009 bronchoscopies from two sites of Charité Universitätsmedizin Berlin—Campus Charité Mitte and Campus Virchow Klinikum—covering the years 2022 and 2023. |
| Participants | 6 | (*a*) *Cohort study*—Give the eligibility criteria, and the sources and methods of selection of participants. Describe methods of follow-up  *Case-control study*—Give the eligibility criteria, and the sources and methods of case ascertainment and control selection. Give the rationale for the choice of cases and controls  *Cross-sectional study*—Give the eligibility criteria, and the sources and methods of selection of participants | 5 | All flexible bronchoscopies performed during the study period were included, irrespective of whether they were conducted without securing the airway or with an endotracheal tube as airway protection. |
|  |  | (*b*) *Cohort study*—For matched studies, give matching criteria and number of exposed and unexposed  *Case-control study*—For matched studies, give matching criteria and the number of controls per case | N/A | Not applicable |
| Variables | 7 | Clearly define all outcomes, exposures, predictors, potential confounders, and effect modifiers. Give diagnostic criteria, if applicable | 6 | Safety was assessed by the occurrence of complications documented in procedure reports and patient records. […] Complications were further classified into the following categories: bleeding, hypoxia, cardiac arrhythmia, hypotonia, pneumothorax, extubation-related complications and others. |
| Data sources/ measurement | 8* | For each variable of interest, give sources of data and details of methods of assessment (measurement). Describe comparability of assessment methods if there is more than one group | 6 | Safety was assessed by the occurrence of complications documented in procedure reports and patient records. |
| Bias | 9 | Describe any efforts to address potential sources of bias | 6 | In cases where multiple complications occurred, only the most clinically significant event per patient was recorded, in order to provide a clear and unbiased assessment of complication rates. |
| Study size | 10 | Explain how the study size was arrived at | 8 | Based on the expected cohort size of approximately 2000 patients and an anticipated complication rate below 5%, the study provides >80% power (one-sided exact binomial test, α = 0.025) to demonstrate that the true complication rate is below 6.5% |

Continued on next page

| Quantitative variables | 11 | Explain how quantitative variables were handled in the analyses. If applicable, describe which groupings were chosen and why | 8 | Descriptive statistics were calculated to evaluate continuous variables, including mean with standard deviation.. Sedation rates and age were compared between the groups using independent samples t-test. |
| --- | --- | --- | --- | --- |
| Statistical methods | 12 | (*a*) Describe all statistical methods, including those used to control for confounding | 8 | To investigate risk factors for bronchoscopy-associated complications, we performed a generalized estimating equations (GEE)-analysis with a binary logistic link, accounting for repeated procedures within patients |
|  |  | (*b*) Describe any methods used to examine subgroups and interactions | 14 | For this analysis, the elderly cohort was further stratified into two subgroups (70–79 years and ≥80 years) to explore potential age-related effects within the elderly population. |
|  |  | (*c*) Explain how missing data were addressed | 8 | Analyses were performed using available data, and cases with missing values were excluded from the respective analyses. |
|  |  | (*d*) *Cohort study*—If applicable, explain how loss to follow-up was addressed  *Case-control study*—If applicable, explain how matching of cases and controls was addressed  *Cross-sectional study*—If applicable, describe analytical methods taking account of sampling strategy | N/A | Not applicable |
|  |  | (*e*) Describe any sensitivity analyses | N/A | Not applicable |
| Results | | | | |
| Participants | 13* | (a) Report numbers of individuals at each stage of study—eg numbers potentially eligible, examined for eligibility, confirmed eligible, included in the study, completing follow-up, and analysed | 8 | In the years 2022 and 2023, 1392 patients underwent 1841 flexible bronchoscopies. 196 patients underwent multiple bronchoscopies (up to 19). 466 bronchoscopies (25.3%) were performed in the elderly group and 1375 bronchoscopies (74.7%) the non-elderly group. |
|  |  | (b) Give reasons for non-participation at each stage | N/A | Not applicable |
|  |  | (c) Consider use of a flow diagram | 24 | Figure 1 |
| Descriptive data | 14* | (a) Give characteristics of study participants (eg demographic, clinical, social) and information on exposures and potential confounders | 8-9 | Table 1: Baseline characteristics |
|  |  | (b) Indicate number of participants with missing data for each variable of interest | 11 | Table 3: Sedation |
|  |  | © *Cohort study*—Summarise follow-up time (eg, average and total amount) | N/A | Not applicable |
| Outcome data | 15* | *Cohort study*—Report numbers of outcome events or summary measures over time | 12-13 | Table 4: Major and minor complications |
|  |  | *Case-control study—*Report numbers in each exposure category, or summary measures of exposure | N/A | Not applicable |
|  |  | *Cross-sectional study—*Report numbers of outcome events or summary measures | N/A | Not applicable |
| Main results | 16 | (*a*) Give unadjusted estimates and, if applicable, confounder-adjusted estimates and their precision (eg, 95% confidence interval). Make clear which confounders were adjusted for and why they were included | 14 | Table 5: Generalized Estimating Equations-analysis – risk factors for bronchoscopy-associated complications |
|  |  | (*b*) Report category boundaries when continuous variables were categorized | 5 | Based on these criteria, we excluded 168 bronchoscopies and formed two groups: bronchoscopies performed on patients that were 70 years or older (elderly group) and bronchoscopies performed on patients younger than 70 years (non-elderly group) at the time of the procedure. |
|  |  | (*c*) If relevant, consider translating estimates of relative risk into absolute risk for a meaningful time period | N/A | Not applicable |

Continued on next page

| Other analyses | 17 | Report other analyses done—eg analyses of subgroups and interactions, and sensitivity analyses | 14 | Table 5: Generalized Estimating Equations-analysis – risk factors for bronchoscopy-associated complications |
| --- | --- | --- | --- | --- |
| Discussion | | | | |
| Key results | 18 | Summarise key results with reference to study objectives | 15 | This large retrospective analysis of 1841 flexible bronchoscopies performed on 1392 patients demonstrates that bronchoscopy is a safe procedure with an overall complication rate of 2.3%, when being performed in a large tertiary care center with dedicated bronchoscopy units. […] Complication rates did not differ significantly between bronchoscopies in elderly (1.7%) and non-elderly patients (2.5%). In addition, the distribution of major and minor complications was also comparable between the age groups, indicating that advanced age alone is not a risk factor. Instead, higher complication risks were associated with specific procedures such as transbronchial forceps biopsy and endobronchial valve implantation. |
| Limitations | 19 | Discuss limitations of the study, taking into account sources of potential bias or imprecision. Discuss both direction and magnitude of any potential bias | 17 | However, certain limitations need to be considered. Due to the retrospective and monocentric design of this study, the results may not be generalizable and carry potential risks for documentation bias. |
| Interpretation | 20 | Give a cautious overall interpretation of results considering objectives, limitations, multiplicity of analyses, results from similar studies, and other relevant evidence | 18 | In our study, flexible bronchoscopy under sedation was safe across all groups with low complication rates. While our findings indicate that invasive interventions, such as transbronchial forceps biopsies and endobronchial valve implantations, are linked to a higher risk of adverse events, age did not heighten procedural risk. |
| Generalisability | 21 | Discuss the generalisability (external validity) of the study results | 17 | Due to the retrospective and monocentric design of this study, the results may not be generalizable and carry potential risks for documentation bias. |
| Other information | |  | | |
| Funding | 22 | Give the source of funding and the role of the funders for the present study and, if applicable, for the original study on which the present article is based | 28 | No funding was received for this study. |

*Give information separately for cases and controls in case-control studies and, if applicable, for exposed and unexposed groups in cohort and cross-sectional studies.

**Note:** An Explanation and Elaboration article discusses each checklist item and gives methodological background and published examples of transparent reporting. The STROBE checklist is best used in conjunction with this article (freely available on the Web sites of PLoS Medicine at http://www.plosmedicine.org/, Annals of Internal Medicine at http://www.annals.org/, and Epidemiology at http://www.epidem.com/). Information on the STROBE Initiative is available at www.strobe-statement.org.
